# Direct conscious telemetry recordings demonstrate increased renal sympathetic nerve activity in rats with chronic kidney disease

**DOI:** 10.3389/fphys.2015.00218

**Published:** 2015-08-04

**Authors:** Ibrahim M. Salman, Divya Sarma Kandukuri, Joanne L. Harrison, Cara M. Hildreth, Jacqueline K. Phillips

**Affiliations:** ^1^Department of Biomedical Sciences, Faculty of Medicine and Health Sciences, Macquarie UniversitySydney, NSW, Australia; ^2^School of Veterinary and Life Sciences, Murdoch UniversityMurdoch, WA, Australia

**Keywords:** renal sympathetic nerve activity, blood pressure, chemoreflex, open-field stress, hypertension, polycystic kidney disease, telemetry

## Abstract

Chronic kidney disease (CKD) is associated with sympathetic hyperactivity and impaired blood pressure control reflex responses, yet direct evidence demonstrating these features of autonomic dysfunction in conscious animals is still lacking. Here we measured renal sympathetic nerve activity (RSNA) and mean arterial pressure (MAP) using telemetry-based recordings in a rat model of CKD, the Lewis Polycystic Kidney (LPK) rat, and assessed responses to chemoreflex activation and acute stress. Male LPK and Lewis control animals (total *n* = 16) were instrumented for telemetric recording of RSNA and MAP. At 12–13 weeks-of-age, resting RSNA and MAP, sympathetic and haemodynamic responses to both peripheral (hypoxia: 10% O_2_) and central chemoreflex (hypercapnia: 7% CO_2_) activation and acute stress (open-field exposure), were measured. As indicators of renal function, urinary protein (U_Pro_) and creatinine (U_Cr_) levels were assessed. LPK rats had higher resting RSNA (1.2 ± 0.1 vs. 0.6 ± 0.1 μV, *p* < 0.05) and MAP (151 ± 8 vs. 97 ± 2 mmHg, *p* < 0.05) compared to Lewis. MAP was negatively correlated with U_Cr_ (*r* = −0.80, *p* = 0.002) and positively correlated with RSNA (*r* = 0.66, *p* = 0.014), with multiple linear regression modeling indicating the strongest correlation was with U_cr_. RSNA and MAP responses to activation of the central chemoreflex and open-field stress were reduced in the LPK relative to the Lewis (all *p* < 0.05). This is the first description of dual conscious telemetry recording of RSNA and MAP in a genetic rodent model of CKD. Elevated RSNA is likely a key contributor to the marked hypertension in this model, while attenuated RSNA and MAP responses to central chemoreflex activation and acute stress in the LPK indicate possible deficits in the neural processing of autonomic outflows evoked by these sympathoexcitatory pathways.

## Introduction

Sympathetic nervous system (SNS) hyperactivity is synonymous with chronic kidney disease (CKD), contributing to hypertension, renal disease progression, and consequent cardiovascular morbidity and mortality (Penne et al., 2009; Grassi et al., 2011). Plasma catecholamine levels (Zoccali et al., 2002; Grassi et al., 2011) and renal noradrenaline spillover (Schlaich et al., 2013) are increased, and direct measurements of muscle sympathetic nerve activity (SNA) reveal elevated tonic levels (Neumann et al., 2007; Grassi et al., 2011; Schlaich et al., 2013). However, despite the various approaches to quantify sympathetic activity in humans and experimental animals, each has inherent limitations. Measurement of plasma catecholamine levels can reveal global activity but not the discrete contributions of organ-specific sympathetic nerve beds and noradrenaline spillover studies, while organ specific, must take into consideration not only SNA but also altered neurotransmitter uptake as an underlying mechanism. Elevated levels of muscle SNA features in many cardiovascular diseases and as a tool has provided an incredibly valuable snapshot into SNA at both the multiunit and single unit recording level in humans (Lambert et al., 2008) and has been used to assess for example the impact of renal denervation on muscle SNA in essential hypertension (Hering et al., 2013), however muscle SNA cannot provide mechanistic data as to what pattern of SNA is influencing visceral organs such as the kidney (Grassi et al., 2015) and it does not provide a long-term monitoring approach. Direct recordings of SNA to specific organ beds can be acquired from acute recordings in unconscious animals (Yao et al., 2015), however these may be unavoidably confounded by the effect of anesthetic on both SNA and blood pressure. Urethane anesthesia, for example, has been shown to both promote and inhibit SNA (Shimokawa et al., 1998; Wang et al., 2014) and altered baseline levels of blood pressure due to anesthetic will alter SNA through baroreflex mechanisms. Many of the reflex autonomic responses tested through pharmacological manipulation of blood pressure may also be influenced by anesthesia. For example responses to the ganglionic blocker hexamethonium have been shown to have different responses on sympathetic control of blood pressure in anesthetized vs. conscious animals, in both normotensive and hypertensive models (Biancardi et al., 2007).

Knowing the baseline level of SNA to a specific target organ can be of critical importance. For example, increased sympathetic outflow to the kidney may have a greater role in the long-term control of blood pressure in CKD, given the role of renal SNA (RSNA) in not only altering blood flow, but also regulating renin secretion and salt and water reabsorption (Johns et al., 2011). This is supported by recent evidence showing that renal denervation, using a catheter-based approach that disrupts renal sympathetic nerves in the adventitia of the renal arteries, can mitigate sympathetic hyperactivity in CKD patients, contributing to not only reductions in blood pressure but also improving renal haemodynamics, enhancing glomerular filtration rate, and reducing albuminuria (Hering et al., 2012; Kiuchi et al., 2013; Schlaich et al., 2013). Our current direct knowledge of RSNA and autonomic reflex control in CKD is still limited however.

The Lewis Polycystic Kidney (LPK) rat is a model of nephronophthisis, a form of autosomal recessive polycystic kidney disease arising from a spontaneous mutation in the *Nek8* gene (McCooke et al., 2012). We have previously demonstrated both indirect and direct evidence for sympathetic overactivity in the LPK. Indirectly, hypotensive responses to ganglionic blockade (Phillips et al., 2007; Ameer et al., 2014), bradycardic responses to β_1_-adrenoceptor blockade (Harrison et al., 2010) and low-frequency power of systolic blood pressure variability (Harrison et al., 2010; Hildreth et al., 2013b) are all increased in the LPK. Directly, we have recently shown that RSNA in anesthetized animals display elevated absolute baseline tonic levels (Salman et al., 2014, 2015; Yao et al., 2015). Given the potential effect of anesthesia on SNA, which may be influenced to a greater degree in the disease state (Hildreth et al., 2013a), a key objective of the present study was to record RSNA in conscious unrestrained animals using telemetry, examining the hypothesis that a sustained elevation in RSNA is a key pathological feature of CKD. We also sought to identify if altered sympathetic activity in the conscious LPK is associated with deficits in regulatory pathways known to impact autonomic neuroregulation of the cardiovascular system including the chemoreceptor reflex and behavioral stress. Tonic activation of excitatory chemoreceptor afferents has been proposed as a driver of elevated SNA in CKD patients (Hering et al., 2007; Despas et al., 2009) and we have recently shown under anesthetized conditions that the LPK animals have impaired peripheral and central chemoreflex responses (Yao et al., 2015). Accordingly, our second aim was to assess RSNA and blood pressure responses to central (hypercapnia) and peripheral (hypoxia) chemoreceptor stimulation in the conscious LPK. Another potential contributor to sympathoexcitation in CKD is altered reactivity to stress, with exaggerated sympathetic responses to acute stress reported in other hypertensive models (DiBona et al., 1995; Head and Burke, 2004; D'Angelo et al., 2006). In CKD however, results are variable, with reports of unchanged (Agarwal et al., 1991) or exaggerated (Seliger et al., 2008) haemodynamic response to mental stressors documented. Therefore, our third aim was to assess sympathetic and blood pressure reactivity to acute stress in the conscious LPK.

## Materials and methods

### Animals

Telemetry probes were implanted in male Lewis (*n* = 13) and LPK (*n* = 9) rats. From the control group, 9 provided successful recordings of both SNA and blood pressure, while from the LPK group, 7 were successful recordings. Those animals that did not provide dual recordings for the duration of the study were removed from the study (final *n* = 16). Animals were kept under a 12-h light/dark cycle and received a standard pellet diet and water *ad libitum*. All studies were approved by the Animal Ethics Committee of Macquarie University and carried out in accordance with the Australian Code of Practice for the Care and Use of Animals for Scientific Purposes (8th Edition, 2013).

### Telemetry probe implantation

At 10–11 weeks of age, rats underwent surgery to implant a telemeter equipped with nerve recording electrodes and arterial catheter (Model TR46SP/56SP, Telemetry Research/Millar, Auckland, New Zealand). Animals were anesthetized with isoflurane (5% in 100% O_2_) for induction and 1–3% for maintenance. Pre-operative pain relief (carprofen 2.5 mg/kg s.c., Norbrook Laboratories, VIC, Australia) and antibiotic (cephazolin 55 mg/kg i.m., Hospira Pty Ltd, VIC, Australia) were administered. An abdominal and dorsal skin incision was made and the nerve recording electrode subcutaneously tunneled from the abdominal through to dorsal incision site. The left kidney was exposed retroperitoneally and a 2-mm portion of the renal nerve, coursing between the abdominal aorta and left renal artery, isolated. The recording electrode was anchored to the aorta and renal artery using non-absorbable 7/0 prolene sutures and the renal nerve gently placed on the electrode and embedded in a silicone elastomer (Kwik-sil®, World Precision Instruments, FL, USA). The ground electrode was sutured to the flank muscles exterior to the dorsal incision site and the dorsal incision closed. The blood pressure catheter was placed by one of two methods depending upon catheter type. Either, the peritoneal cavity was exposed and the catheter (Millar type/TR56SP) was inserted into the abdominal aorta, such that the tip of the catheter was distal to the renal artery. The catheter was secured in place using a plastic mesh (Telemetry Research) and cyanoacrylate cement (Histoacryl®, B Braun, NSW, Australia), and lidocaine (1%, Pfizer, NSW, Australia) applied to induce vasodilation and improve hind limb blood flow. Alternatively, the blood pressure catheter (fluid-filled type/TR46SP) was placed into the femoral artery with the probe body placed within the peritoneal cavity as we have described previously (Hildreth et al., 2013b). Supplemental fluids were provided (saline, 6 ml/kg i.p.) and the peritoneal cavity closed. All skin incisions were closed using wound closure clips. Following cessation of anesthesia, post-operative pain relief was provided [buprenorphine (Temgesic®, Reckitt Benckiser, NSW, Australia, 50 μg/kg s.c.). Rats were allowed to recover for at least 1 week in order to re-establish circadian rhythms (Hildreth et al., 2013b). Pain relief (carprofen, 2.5 mg/kg, s.c. and/or buprenorphine, 50 μg/kg s.c.) and supplemental fluid therapy (up to 60 ml/kg/day 0.9% saline and/or 5% glucose s.c.) were administered as required.

### Experimental protocol

All protocols were carried out when animals were aged between 12 and 13 weeks over a ~7-day period, allowing a minimum 1 week post-surgical recovery period for reestablishment of circadian rhythms, adequate wound healing and increase in body weight and food and water intake. Animals were individually housed and RSNA and blood pressure data acquired using a receiver (TR162 or TR180: Telemetry Research/Millar) interfaced with a CED Micro 1401 data acquisition system (Cambridge Electronic Designs Ltd, Cambridge, UK). A battery charging pad (TR802/TR180 Telemetry Research/Millar) was placed under each individual animal cage. The blood pressure signal was sampled at a minimum of 500 Hz and RSNA at 2 kHz and continuously displayed on Spike 2 software (v7, CED Ltd., Cambridge, UK). The original RSNA signal was amplified, filtered between 50 and 2000 Hz, full-wave rectified and integrated (1 s smoothing constant). All experiments were conducted between 9:00 a.m. and 5:00 p.m. Each rat underwent no more than one study per day. Resting data was collected at the beginning of the experimental period. Chemoreflex and stress response experiments (see below) were then performed in random order. Ganglionic blockade was the last protocol undertaken. Note not all animals contributed to the data set for each protocol.

#### Resting data

Blood pressure and RSNA were recorded for 5 min every 15 min from 9:00 a.m. to 5:00 p.m. over two consecutive days. No other intervention was undertaken on these days.

#### Chemoreceptor reflex

Animals were placed in a custom-made plexiglass chamber to which they were previously acclimatized. The chamber was initially filled with medical grade air [21% O_2_ balance N_2_ (BOC Ltd, NSW, Australia), 0.5–1 L/min] and O_2_ and CO_2_ levels continuously monitored (CapStar-100 CO_2_ analyser®, CWE Inc., Ardmore, PA, USA and Gas analyser®, ADInstruments Pty Ltd, NSW, Australia). Once the animal was resting quietly, as indicated by stable measurements of mean arterial pressure (MAP) and RSNA, a 5-min baseline recording was obtained. Following this, the chamber was flushed with either a hypoxic (10% O_2_ balance N_2_, BOC Ltd,) or hypercapnic (7% CO_2_ balance O_2_, BOC Ltd,) gas mixture to activate the peripheral and central chemoreceptors, respectively. Approximately 15 s were required to reach the target concentration of O_2_ (10%) and CO_2_ (7%) within the chamber. Reducing inspired O_2_ from 21 to ~10% in conscious rats has been shown previously to effectively reduce PaO_2_, but not PaCO_2_, while increasing inspired CO_2_ from 0 to ~7% results in an increase in PaCO_2_without a change in PaO_2_(Pepelko and Dixon, 1975). Animals were exposed to these gas mixtures for no more than 5 min, following which the chamber was flushed with medical grade air. Following a 10–20 min recovery period, another 5 min baseline recording was obtained and the chamber was filled with the alternate gaseous mixture and MAP and RSNA recorded for another period of 5 min. The order of gas exposure was randomized and at the end of the experiment, the rat was returned to its home cage.

#### Open-field stress

Baseline levels of MAP and RSNA were recorded for 10 min while the animal was in its home cage. The animal was then gently transferred into a brightly lit open field (~90 cm diameter circular chamber with 40 cm wall) and the MAP and RSNA recorded for 40 min. The animal was then returned to its home cage.

#### Ganglionic blockade

Baseline MAP and RSNA was recorded for 5 min while the animal was in its home cage. The ganglionic blocker, hexamethonium (20 mg/kg s.c., Sigma Aldrich, NSW, Australia) was then administered and MAP and RSNA recorded for at least 30 min. The peak response to hexamethonium however was seen in under 10 min in both strains.

### Indicators of renal function

Animals were individually held in metabolic cages (Tecniplast, NSW, Australia) for 24 h to collect urine samples and determine 24 h urine production and water consumption. Urine was then centrifuged at 3000 rpm for 5 min and stored at −20°C until further assayed for urinary protein (U_Pro_) and creatinine (U_Cr_) using an IDEXX VetLab analyser (IDEXX Laboratories Pty Ltd., NSW, Australia).

### Euthanasia

After all protocols had been undertaken, rats were euthanased with an overdose of 60 mg/kg sodium pentobarbital i.p. (Lethabarb Euthanasia®, Virbac Pty Ltd, NSW, Australia) and death levels of RSNA recorded.

### Data analysis

**Figure 1:**
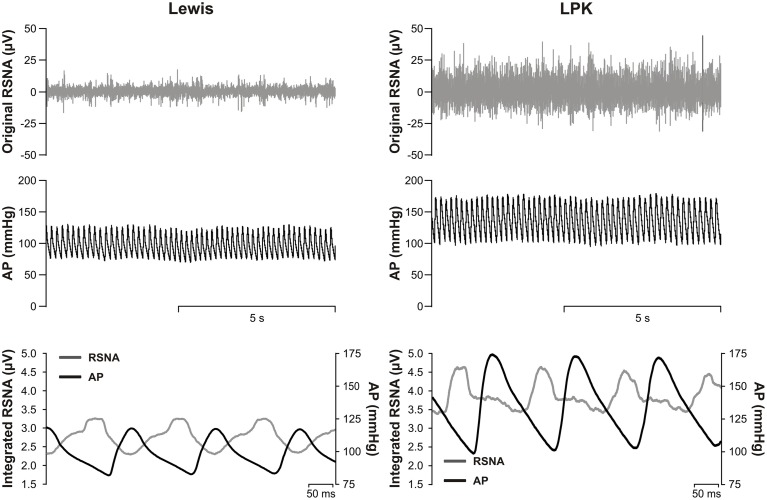
**Top, representative raw data traces of original renal sympathetic nerve activity (RSNA) and arterial pressure (AP) from conscious Lewis (left) and Lewis Polycystic Kidney (LPK, right) rats**. Bottom, pulse modulation of RSNA using pulse triggered averages of AP and integrated RSNA (solid lines) from the same Lewis (left) and LPK (right) animals. Note the inverse phasic relationship between AP and RSNA in the pulse-triggered averages, typical of pulse modulation of SNA.

All data was analyzed offline using Spike 2 software and GraphPad Prism (GraphPad Prism software v6 Inc., La Jolla, CA, USA). Background nerve activity acquired following ganglionic blockade was subtracted from all RSNA recordings. The level of nerve activity following ganglionic blockade was compared against that obtained following euthanasia and the quality of nerve activity verified by assessing the pulse modulation of RSNA (Figure 1) as described previously (Guild et al., 2012; Stocker and Muntzel, 2013).

#### Resting data

From the MAP and RSNA recording taken between 9:00 a.m. to 5:00 p.m., a 3-h period, where no signal dropout was observed was identified for each animal. Care was taken to ensure that the time of data analysis was not biased toward morning vs. afternoon in the Lewis vs. LPK, and thus the 3-h periods were randomly distributed. Blood pressure and RSNA was averaged over each 3-h period over the two consecutive days to create one estimate for each variable per animal.

#### Chemoreflex data

To temporally capture changes in the chemoreflex response, blood pressure, and nerve responses were averaged into 1-min bins. The level of RSNA, determined during the 1 min period immediately prior to exposure to either hypoxic or hypercapnic gas mixture was set as 100%. Maximum changes in RSNA (μV) relative to the 1 min baseline were also measured. Changes in MAP were expressed relative to the level of MAP 1 min prior to chemoreflex activation.

#### Open-field stress

The response to exposure to the open field chamber was evident in less than 10 min after initial exposure and consequently, only the first 10 min of data acquired was analyzed. To temporally capture changes during the open-field exposure, blood pressure, and nerve responses were averaged into 2-min bins. The averaged level of MAP and RSNA determined during the 2 min period immediately prior to exposure to the open-field was set as 100% and changes then expressed relative these baseline levels.

#### Ganglionic blockade

Maximum MAP and RSNA responses to hexamethonium were measured relative to a 5-min baseline of these variables.

#### Correlation of MAP with indicators of renal function and RSNA

In order to determine if there was any relationship between MAP and the indicators renal function [U_Pro_, U_Cr_ and urinary protein: creatinine ratio (UPC)] and/or RSNA, and the relative strength of the relationships, multiple linear regression modeling was undertaken, using the IBM Statistical Package for the Social Sciences (SPSS; v22, IL, USA). MAP was set as the dependent variable and U_Pro_, U_Cr_, UPC, and RSNA as the independent variables, using a stepwise selection method of entry and pairwise exclusion of missing values. Correlation analysis [Pearson correlation coefficient (r) and significance (1-tailed)] and results of the full model [adjusted R^2^ value, being the relative predictive powers of the model adjusted for degrees of freedom), significance and β-standardized regression coefficient (β) for the entered independent variables] are provided. *n* = Number of data pairs.

### Statistical analysis

All data are expressed as mean ± standard error of the mean (SEM). Statistical analysis was performed using GraphPad Prism (GraphPad Prism software v6 Inc.). A Brown-Forsythe test was used to determine if there were any differences in the variance, and if so, the data was log-transformed before statistical analysis. Baseline levels of MAP and RSNA, renal function, maximum RSNA (μV) responses to chemoreflex activation and open-field exposure, responses to ganglionic blockade and stability of RSNA were analyzed between strains using a two-tailed Student's *t*-test. The effect of chemoreflex activation or stress on the level of MAP and RSNA was identified within each strain using a repeated measures One-Way ANOVA. Any strain differences in the response to chemoreflex activation or stress were identified using a Two-Way ANOVA. Significance was defined as *p* ≤ 0.05.

## Results

### Phenotypic characteristics of Lewis and LPK rats

**Figure 2:**
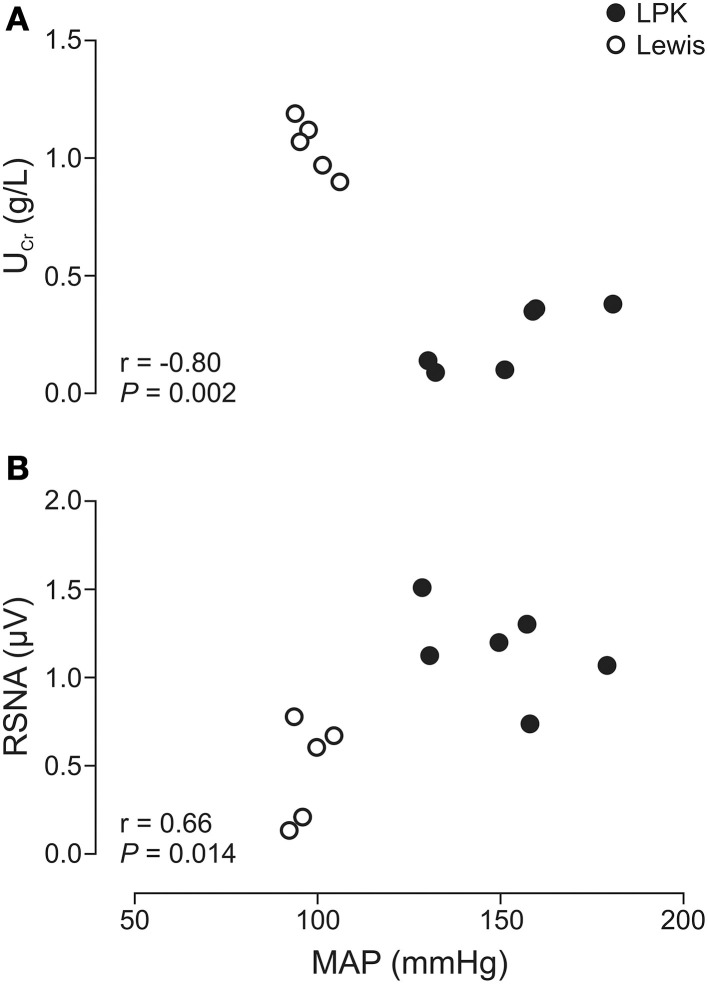
**Correlations of mean arterial pressure (MAP) vs. (A) urinary creatinine (U_Cr_) and (B) renal sympathetic nerve activity (RSNA), in Lewis and Lewis Polycystic Kidney (LPK) rats derived from multiple linear regression (*n* = 11)**. Pearson correlation coefficient (r) and significance are provided for each relationship within the figure panel.

Daily water intake and urine output, U_Pro_, UPC, MAP, and resting absolute RSNA were significantly higher in the LPK rats vs. Lewis controls (Table 1). U_Cr_ excretion levels were significantly lower in the LPK compared with Lewis (Table 1). MAP was significantly negatively correlated with U_cr_(Figure 2A) and positively correlated with RSNA (Figure 2B). In the multiple linear regression model however, U_cr_ was the only significant predictor variable (*n* = 11 data pairs, adjusted *R*^2^ = 0.595, β coefficient = −0.797, *p* = 0.003), indicating a stronger relationship between MAP and U_cr_ than MAP and RSNA. U_Pro_ and UPC were not significantly correlated with MAP and were both excluded from the final model.

**Table 1:** **Phenotypic characteristics of Lewis and LPK rats**.

**Parameter_n_**	**Lewis/8**	**LPK/6**	***p*-value**
Body weight (g)	332 ± 8	254 ± 8	< 0.0001
Water intake (ml/24 h)	25 ± 1	50 ± 3	< 0.0001
Urine output (ml/24 h)	11 ± 1	51 ± 3	< 0.0001
U_Pro_ (g/L)	0.05 ± 0.01	0.89 ± 0.41	< 0.0001
U_Cr_ (g/L)	1.2 ± 0.2	0.2 ± 0.1	0.0006
UPC	0.06 ± 0.02	5.9 ± 3.2	< 0.0001
MAP (mmHg)	97 ± 2	151 ± 8	< 0.0001
RSNA (μV)	0.6 ± 0.1	1.2 ± 0.1	0.0190

LPK, Lewis Polycystic Kidney; U_Pro_, urinary total protein; U_Cr_, urinary creatinine; UPC, urinary protein-to-creatinine ratio; RSNA, renal sympathetic nerve activity and MAP, mean arterial pressure. Results are expressed as mean ± SEM. In the Lewis, UPC was not calculated in 3/9 animals due to lack of detectable protein levels in the urine. Accordingly, only 6 Lewis animals were used to compare mean values of UPC. p-values determined using a two-tailed Student's t-test. (n) values denoted in subscript and represent the minimum number in each group.

### Responses to chemoreceptor reflex activation

#### Peripheral chemoreflex activation

**Figure 3:**
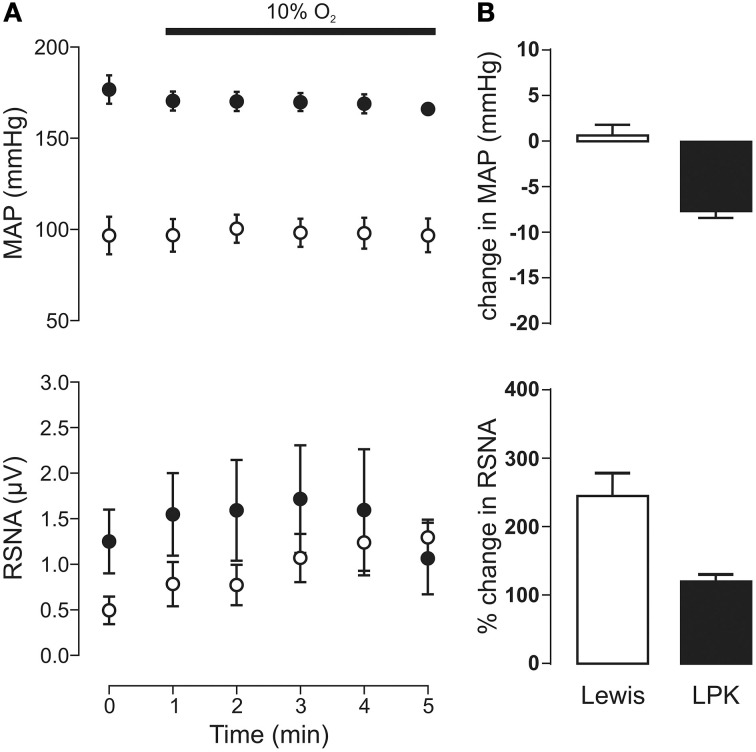
**Mean arterial pressure (MAP) and renal sympathetic nerve activity (RSNA) responses to peripheral chemoreflex activation in Lewis and Lewis Polycystic Kidney (LPK) rats**. **(A)** Data points are 1-min averages of MAP and RSNA measured for 1 min before (time zero) and 5 min during a hypoxic challenge (10% O_2_). Results are expressed as mean ± SEM. Minimum *n*/group = 4. **(B)** Average change in MAP and RSNA during the challenge. Results are expressed as mean ± SEM.

Exposure to hypoxia (Figure 3) did not significantly change MAP in either the LPK or Lewis (both *p* ≥ 0.05). A trend for an overall increase in RSNA in response to hypoxia was evident, most notably in the Lewis animals (Figure 3B), however, Two-Way ANOVA indicated no significant effect for either strain (*p* = 0.29) or time (*p* = 0.15) nor was there an interaction between strain and time (*p* = 0.13, *n* = 9).

#### Central chemoreflex activation

**Figure 4:**
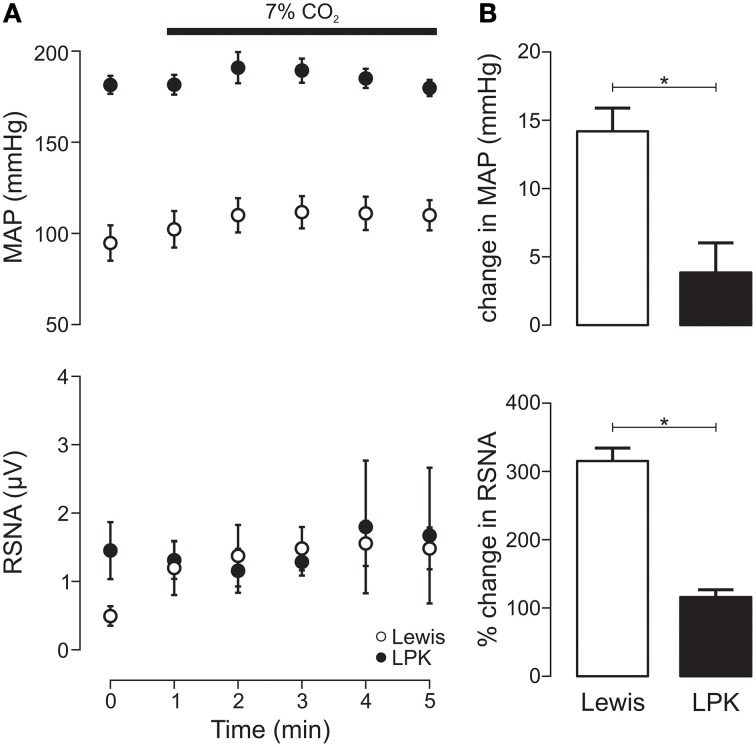
**Mean arterial pressure (MAP) and renal sympathetic nerve activity (RSNA) responses to central chemoreflex activation in Lewis and Lewis Polycystic Kidney (LPK) rats**. **(A)** Data points are 1-min averages of RSNA and MAP measured for 1 min before (time zero) and 5 min during a hypercapnic challenge (7% CO_2_). **(B)** average change in MAP and RSNA during the challenge. Results are expressed as mean ± SEM. ^*^p < 0.05. Minimum *n*/group = 4.

Exposure to hypercapnia produced an increase in MAP (*p* = 0.005) alongside a trend toward increasing RSNA (*p* = 0.06) in the Lewis (Figure 4A). In the LPK, no change in MAP (*p* = 0.10) or RSNA (*p* = 0.68) was observed (Figure 4A). Consequently, both the pressor and sympathoexcitatory response to exposure to hypercapnia was greater in the Lewis compared with the LPK (both *p* < 0.05, Figure 4B).

### Responses to acute open-field stress

**Figure 5:**
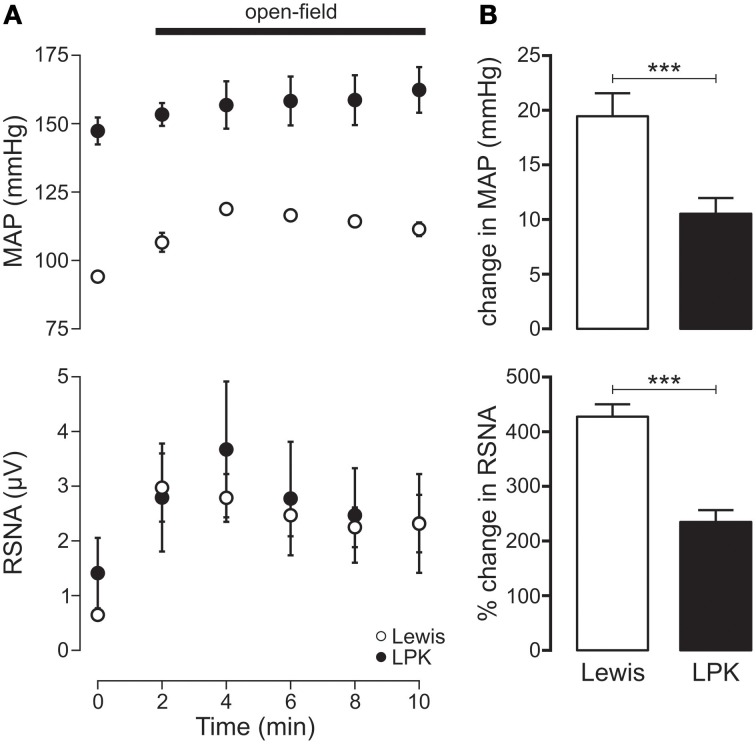
**(A)** Mean arterial pressure (MAP) and renal sympathetic nerve activity (RSNA) responses to acute open-field stress in Lewis and Lewis Polycystic Kidney (LPK) rats. Data points are 2-min averages of RSNA and MAP measured for 2 min before (time zero) and the first 10 min during acute stress exposure. **(B)** Average change in MAP and RSNA during the first 10 min of the acute stress exposure. Results are expressed as mean ± SEM. ^***^p < 0.001. Minimum *n*/group = 6.

Exposure to the open-field environment produced an increase in both MAP (*p* < 0.001) and RSNA (*p* = 0.001) in the Lewis (Figure 5A). In the LPK, only an increase in RSNA (*p* = 0.02) was observed (Figure 5A). Consequently, both the pressor (*p* = 0.003) and sympathoexcitatory (*p* < 0.001) response to exposure to the open-field environment was greater in the Lewis compared with LPK (Figure 5B).

### Responses to ganglionic blockade

Administration of hexamethonium produced a fall in MAP and RSNA in both the Lewis [RSNA: 2.1 ± 0.1 vs. 1.5 ± 0.2 μV (*n* = 8); MAP: 105 ± 3 vs. 49 ± 4 mmHg (*n* = 7), baseline vs. hexamethonium; all *p* < 0.05] and LPK [RSNA: 3.0 ± 0.2 vs. 1.6 ± 0.2 μV (*n* = 4), MAP: 157 ± 5 vs. 56 ± 6 mmHg (*n* = 4), baseline vs. hexamethonium; all *p* < 0.05]. The absolute magnitude of the fall was significantly greater in the LPK compared to Lewis for MAP (−1.5 ± 8 vs. -56 ± 3 mmHg) and RSNA (−1.3 ± 0.3 vs. −0.6 ± 0.1 μV) (respectively, both *p* < 0.05). The level of RSNA recorded following administration of hexamethonium was not different between Lewis and LPK and in both groups was comparable to that recorded following euthanasia [Lewis: 1.5 ± 0.2 (*n* = 8) vs. 1.3 ± 0.2 (*n* = 9) μV and LPK: 1.6 ± 0.2 (*n* = 4) vs. 1.3 ± 0.2 (*n* = 7) μV, hexamethonium vs. euthanasia; all *p* > 0.05].

## Discussion

This is the first description of conscious concurrent telemetric recordings of RSNA and blood pressure in a genetic rodent model of CKD. The major novel findings of the present study are: (1) LPK rats have sustained elevated absolute RSNA under conscious conditions compared to Lewis control animals; (2) MAP is most strongly correlated with urinary creatinine as an indicator of renal function across both strains of rat, as well as showing a correlation with RSNA, (3) conscious LPK show reduced RSNA and MAP responses to central chemoreceptor reflex activation and acute stress, indicating possible deficits in the neural processing of autonomic outflows evoked by these typically sympathoexcitatory pathways. Together, this shows that in CKD, sympathetic overdrive, as assessed by direct conscious recording of RSNA, is an archetypal feature, likely contributing to the maintained hypertensive state, and there is altered reactivity to reflexogenic and stressful stimuli.

In our study, we used absolute microvolt measures to report baseline RSNA, and percentage change scores to assess autonomic reflex responses. The reporting of SNA is a methodological consideration, as it has been suggested that absolute levels of SNA in voltage units cannot be compared between animals, as SNA level is dependent upon conditions at the recording site, the number and size of the nerve fibers and the proximity of the active fibers to the electrode (Burke et al., 2011), which increases variability between different nerve recordings. Normalizing nerve activity data by setting baseline to 100% and reporting percentage change scores is proposed to avoid any bias that would therefore otherwise arise, and allow for significant differences to be detected in smaller groups of rats. This method, however, does not allow for comparison of baseline activity or maximal changes in absolute units (Burke et al., 2011). This has led some to suggest that reporting properties of nerve activity using both normalized responses and raw rectified voltage is extremely valuable (Huber and Schreihofer, 2010).

The use of telemetry to directly record SNA in conscious animals over a sustained period overcomes many of the limitations associated with other direct and indirect methods of determining SNA. It does however have inherent technical challenges. The surgical procedure and placement of the telemetry probe is limited by animal size, with >200 grams being the minimum bodyweight for probe implantation. This limits the age range of animals that can be studied, which can be confounded by disease if that is a factor that reduces an animal's body weight further. Another key challenge is the ability to determine sympathetic activity from background or artifact related noise (Stocker and Muntzel, 2013). In this study, we obtained successful blood pressure and RSNA recordings from 16 out of 22 (~70%) of animals implanted with probes, with the validity of our recordings confirmed by the burst like activity evident within the recordings, strong coupling of the SNA to the cardiac cycle as evidenced by the pulse modulation of SNA, and the effective elimination of the response after ganglionic blockade with hexamethonium, indicative of the postganglionic nature of the signal (Stocker and Muntzel, 2013). Recordings for a prolonged period require reliable battery capacity and charging of the battery *in-vivo* can be problematic, however, this has recently been greatly facilitated by the use of individual recharging pads optimized to fit a standard rodent cage, thereby allowing for continual home cage charging operation. Other technical difficulties experienced included: signal dropout, which influenced the recording period over which we could obtain resting data; activity-induced signal noise; and the ease of data analysis, which for our study required the generation of custom-made scripts to analyse the data in our chosen software package.

Our work here confirms our previous studies demonstrating evidence of increased sympathetic activity in the LPK, as measured by other direct and indirect approaches (Phillips et al., 2007; Harrison et al., 2010; Hildreth et al., 2013b; Salman et al., 2014; Yao et al., 2015). This includes our confirmation of previous findings that the LPK display enhanced depressor responses to ganglionic blockade (Phillips et al., 2007; Ameer et al., 2014), another indicator of increased sympathetic vasomotor tone that as a methodology has been the subject of some debate (Moretti et al., 2009). Clinically, elevated SNA in CKD patients is of marked significance being associated with all-cause mortality and nonfatal cardiovascular events (Penne et al., 2009), left ventricular hypertrophy (Guízar-Mendoza et al., 2006) and vascular damage (Bruno et al., 2012). While not indicative of any causal relationship, our observation of blood pressure being strongly correlated with urinary creatinine, as an indicator of declining renal function, as well as having a positive association with RSNA could offer insight to a potential mechanism underlying the blood pressure lowering and renoprotective effects reported after renal denervation in CKD patients (Hering et al., 2012; Kiuchi et al., 2013; Schlaich et al., 2013) and animal models (Eriguchi et al., 2015).

Whether the RSNA measures that we have observed in the LPK model reflect SNA to other vascular beds remains unknown. We have recently shown that in the anesthetized preparation, simultaneous recordings of lumbar, renal, and splanchnic nerves in the LPK demonstrate higher baseline absolute levels of SNA (μV) when compared to Lewis controls (Yao et al., 2015). Future, studies in conscious animals are required to verify this. Current evidence suggests that overall activity of the SNS cannot be simply judged from the recording of a single sympathetic nerve bed (Knuepfer and Osborn, 2010). For example, in Dahl hypertensive rats, targeted ablation of the renal nerves and/or the splanchnic nerves independently influenced MAP, with the greatest decline in MAP seen in those rats that underwent both ablations (Foss et al., 2013), suggesting distinct roles for both the renal and splanchnic sympathetic outflows in driving hypertension in this model. In contrast, in an angiotensin II-high salt induced hypertension model, RSNA decreased over the study period, while lumbar SNA did not change (Yoshimoto et al., 2010) and that SNA to either vascular bed was not critical in the pathogenesis of angiotensin II-salt hypertension.

We have previously demonstrated that elevated sympathetic drive in the LPK is associated with an inability to effectively restrain SNA by the baroreflex (Salman et al., 2014) and that peripheral and central chemoreflex responses are blunted in these animals (Yao et al., 2015). An objective of the present work was to assess if the LPK display comparable chemoreflex abnormalities in the conscious state and to test a psychological stressful stimulus, being another reflex response known to modulate SNA (van den Buuse et al., 2001). In contrast to our anesthetized study, activation of the peripheral chemoreflex pathway using hypoxia did not cause a change in SNA or MAP in either the Lewis or LPK animals. This may be because the exposure was not of sufficient time frame and/or in the conscious state the animals were able to respond with a reflex increase in ventilation. Additionally, and with specific reference to the LPK animals, the peripheral chemoreceptors may be already maximally activated. In human studies, in order to test if tonic activation of excitatory chemoreceptor afferents contributes to elevated SNA in patients with CKD, the effect of chemoreflex deactivation on SNA is instead tested, comparing the inhibitory effects of 100% oxygen with that of breathing room air on MSNA and blood pressure (Hering et al., 2007). We will require future experiments using alternative experimental paradigms to investigate the effect of stimulation of the peripheral chemoreflex on RSNA in the conscious state in our CKD model. In the CKD animals however, the central chemoreflex response demonstrated an attenuated sympathoexcitatory and pressor response to hypercapnia when compared to the Lewis control animals. This is directly comparable to our studies in the anesthetized animals (Yao et al., 2015). The mechanisms underlying these effects were not examined in this study, however it is possible that tonic activation of the central chemoreflex pathway in CKD may impair the ability of this reflex to drive further increases in SNA.

Like chemoreflex activation, altered cardiovascular responses to emotional stress have been implicated in the setting of high blood pressure (Ming et al., 2004) and are often evident in different models of hypertension (DiBona and Jones, 1995; Head and Burke, 2004; D'Angelo et al., 2006). The principal response to a stressful stimulus involves the activation of the SNS, release of catecholamines from the adrenal medulla and activation of the hypothalamic-pituitary-adrenocortical axis (Fontes et al., 2011), mechanisms which may become altered during disease. Comparable to our central chemoreflex response, we observed diminution of the sympathetic responses to acute stress in the LPK relative to Lewis, such that the acute rise in RSNA was attenuated and no change in blood pressure was noted. In CKD, previous studies have shown that sympathetic reactivity to mental stress was either unchanged (Agarwal et al., 1991) or exaggerated (Seliger et al., 2008). In these studies, however, sympathetic reactivity was inferred indirectly from assessing differences in the haemodynamic response to stress in test subjects. To our knowledge, this is the first report to show sympathetic responses to stress in CKD using direct recording of both SNA and blood pressure. It is possible that the attenuated sympathetic response and absence of a blood pressure response in the LPK may have been the result of an effective activation of the buffering capacity of the baroreflex; however we believe this is unlikely because we have shown previously that baroreflex control of RSNA and heart rate are markedly impaired in the LPK at this age (Salman et al., 2014). Alternatively, it may indicate RSNA is maximally increased in the LPK, such that it cannot be increased further, or that in the LPK there is an underlying upregulation of endogenous stress pathways (Armitage et al., 2012). Either way, when this response and/or pathway are activated by external factors, the net increase to maximal response is proportionally less than in the control animals.

In conclusion, direct conscious recording of SNA in undisturbed freely moving rats overcomes many of the limitations that other indirect or direct assessments of sympathetic activity are associated with. In a conscious rodent model of CKD, we describe for the first time direct telemetric recording of SNA to the kidney and provide evidence that high blood pressure is significantly correlated with increased RSNA and reduced renal function. The study further shows that the renal disease is associated with both reduced central chemoreflex and stress-induced regulation of RSNA and MAP, indicating that the ability of the CNS to regulate sympathetic outflow and blood pressure is compromised in CKD, therefore emphasizing the complexity of this pathological condition. Findings from the present study are relevant to better understanding the complex nature of this global clinical problem and novel therapies such as the targeting of sympathetic activity to specific organs to limit cardiovascular disease in CKD.

## Author contributions

IS Contributed to design of research, performed experiments; analyzed data; interpreted results of experiments; prepared figures; drafted manuscript; approved final version of manuscript. DK Assisted in the undertaking of experiments and approved final version of manuscript. JH Contributed to the design of research, performed preliminary experiments and approved final version of manuscript. CH Contributed to experimental design, analyzed data, interpreted results of experiments; edited and revised manuscript; prepared figures and approved final version of manuscript. JP–Conception and design of research; analyzed data; interpreted results of experiments, edited and drafted manuscript and responsible for submission of manuscript.

### Conflict of interest statement

The authors declare that the research was conducted in the absence of any commercial or financial relationships that could be construed as a potential conflict of interest.
